# Nutritional Composition and Phytochemical Changes in Wild Melon (*Cucumis melo* var. *agrestis*) Induced by Drying, Frying, and Cooking

**DOI:** 10.1111/1750-3841.70229

**Published:** 2025-05-19

**Authors:** Saghir Ahmed Sheikh, Aasia Akbar Panhwar, Parkash Meghwar, Isaac Duah Boateng

**Affiliations:** ^1^ Department of Applied Sciences Hamdard University Karachi Pakistan; ^2^ Institute of Food Sciences and Technology Sindh Agriculture University Tandojam Sindh Pakistan; ^3^ Department of Food Science and Technology University of Karachi Karachi Pakistan; ^4^ Certified Group 199 W Rhapsody Dr San Antonio, Texas USA

**Keywords:** *Cucumis melo*, drying methods, organic acids, phytoconstituents, wild melon

## Abstract

This study investigated the impact of various processing methods sun drying, dehydration, frying and cooking) on nutritional and phytochemical profile of wild melon (*Cucumis melo* var. *agrestis*). Proximate analysis revealed that drying methods enhanced the concentration of essential nutrients, including carbohydrates, proteins, and fibre, due to moisture reduction. Frying led to an increase in fat content but reduced specific heat‐sensitive vitamins and antioxidants. Cooking resulted in varying phytochemical changes, with some compounds degrading while others became more bioavailable. Notably, sun drying and dehydration preserved higher levels of phenolic compounds and flavonoids, enhancing the antioxidant potential of wild melon. These findings highlight the impact of processing on nutrient retention and phytochemical stability, underscoring the need for optimized techniques to maximize its nutritional benefits. Further research is recommended to assess these nutrients' bioavailability and functional health benefits.

**Practical Application**: Through a systematic examination of different processing methods, the research provides novel insights into how these methods influence the melon's nutritional composition and phytochemistry. The findings demonstrate that sun‐drying optimizes proximate composition, while thermal drying enhances mineral composition. Additionally, the study highlights the preservation of bioactive compounds in fresh samples and the concentration of organic acids in dried samples. This work advances the field of food science by offering a foundation for optimizing processing methods to enhance traditional foods' nutritional and phytochemical profiles, with significant implications for food fortification, preservation, and security.

**Abbreviations**
AAS: atomic absorption spectrophotometer
ANOVA: analysis of variance
AOAC: Association of Officials of Analytical Chemists
DM: dry matter
DMRT: Duncan's multiple range test
GAE: Gallic acid extract
IU: international unit
NFE: nitrogen‐free extract
PCA: principal component analysis
RDA: recommended daily allowance.

## 1 Introduction

Wild melon belongs to the Cucurbitaceae family and is consumed as a vegetable in its pre‐mature phase and as a fruit in its mature stage in rural parts of Pakistan, Afghanistan, India, Malaysia, Iran, and China (Sharma et al. 2024). Wild melon is an inexpensive source of numerous health benefits, including anti‐diabetic, antioxidant, anti‐ulcer, anti‐inflammatory, anti‐angiogenic, anti‐hypothyroidism, and anti‐bacterial activities, due to its rich bioactive compounds (Swamy and Nath 2020; Khalid et al. 2021). Locally identified as *Chibhar*, the fruit is characterized by its oval globose, ellipsoid shape with dark green lines and its raw form is traditionally used for stomach therapy and inducing vomiting (De and De 2020). When ripe, it turns pale yellow with bitter pulp and white seeds, known for their drastic purgative properties (Vieira et al. 2020). However, because of the extremely high moisture content (> 80%), they are classified as highly perishable foods, and suitable processing can reduce their loss.

In rural areas, wild melon is often preserved using traditional methods like sun drying, allowing consumption throughout different seasons. This practice enhances its medicinal value, as the seed‐free pulp aids in relieving nausea and purging (Al‐Snafi 2021). Wild melon's nutritional and phytochemical composition contributes significantly to the health status of rural and urban communities in Sindh, Pakistan, providing a valuable source of macro and micro‐nutrients and medicinal benefits. Using non‐conventional vegetables like wild melon can play a crucial role in improving food security and life expectancy, particularly in regions facing food scarcity and malnutrition (Odukoya et al. 2021). Namet et al. (2023) highlighted the potential of melon and its by‐products in improving food supply and security, emphasizing the importance of exploring wild plants as functional foods to support malnourished communities. To enhance food functionality, foods are usually exposed to different processing methods (thermal, non‐thermal, hybrid etc.). These thermal processing (blanching, boiling, roasting, and frying), pressure (high‐pressure and high‐pressure micro fluidization), and irradiation (high voltage, pulsed electric fields etc.) affect nutritional and bioactive constituents (Boateng and Yang 2021; Boateng 2023; Zhang et al. 2020). Throughout past eras, dehydration has been considered an ideal method of preserving foods to retain nutrients and quality. Frying is one of the most common and traditional procedures in the food industry to produce high‐quality goods with distinct sensory qualities (Zhang et al. 2020; Rani et al. 2023). Drying is a commonly used food preservation, and various drying techniques such as conventional (hot air drying and vacuum drying) and advanced drying (microwave, pulsed vacuum, and infrared drying) have been used to develop desirable food products and increase bioactivity (Boateng 2023). This research focuses on the diverse processing techniques and their effects on wild melon's phytochemical and nutritional properties, an underutilized vegetable with significant health benefits. Besides, despite wild melon's potential, there is a lack of comprehensive studies on how different processing methods influence its nutritional composition and medicinal properties of wild melon.

This study addresses this gap by providing a detailed analysis (proximate analysis, minerals, organic acids, vitamins and phytochemicals) of the impact of different processing methods on wild melon, offering insights that could enhance the utilization of this non‐traditional vegetable to combat malnutrition, hunger, and illness in vulnerable populations (Ali and Bhattacharjee 2023).

## 2 Materials and methods

### 2.1 Procurement of Wild Melon

Wild melon samples were collected from Mithi, Tharparkar (24°74'0N 69°80'0E) (a tropical and sub‐tropic agriculture‐based region with dominant farming shortly after monsoon rainfall), Sindh's local agriculture farms in October‐November 2022. The samples were further certified as authentic samples of wild melon (*Cucumis melo* var. *agrestis*) by a botanist and the herbarium number was SLUBGH 147.

### 2.2 Traditional Processing

**FIGURE 1:**
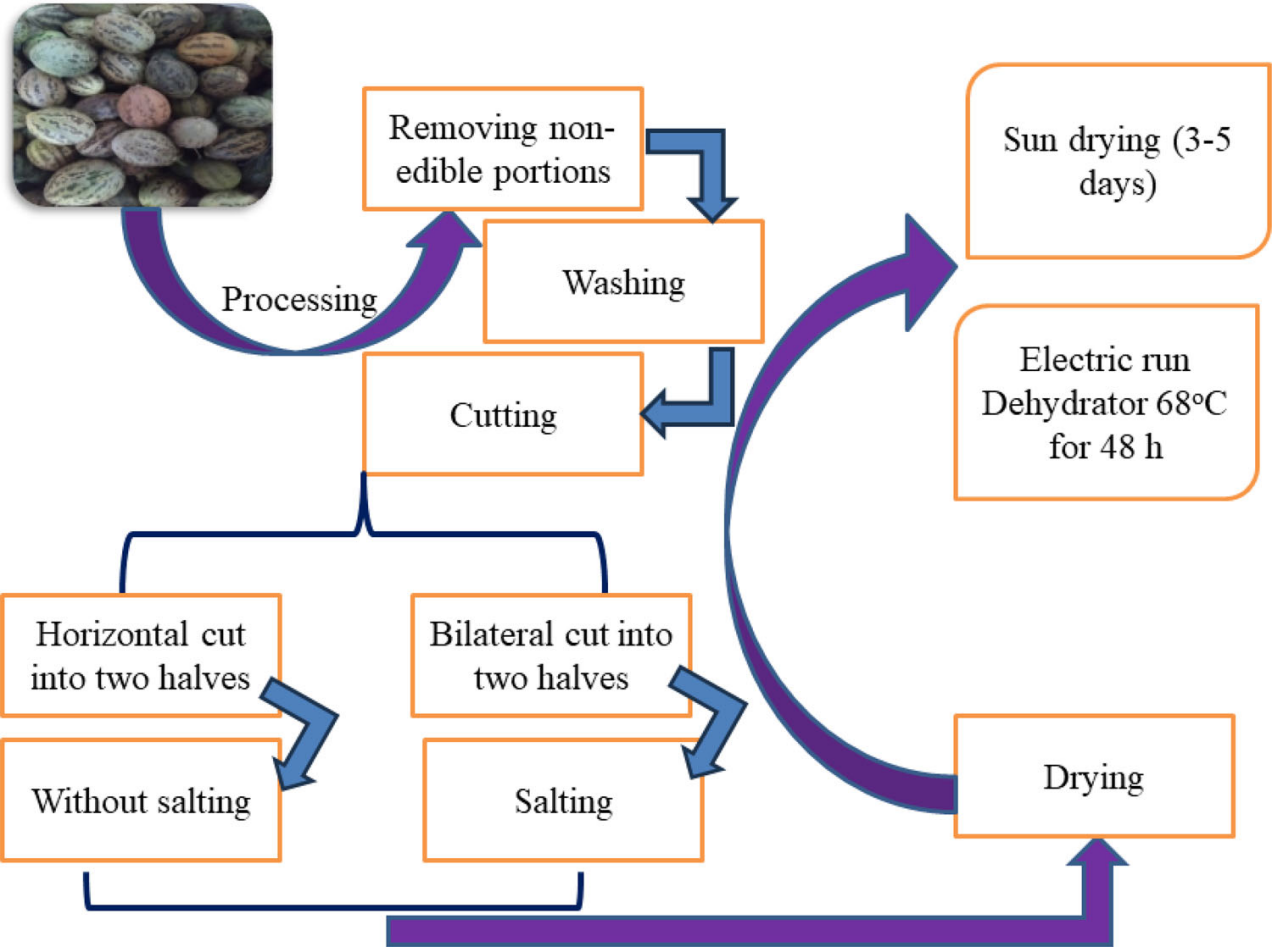
Traditional preservation of *Chibhar* done through drying methods.

The fresh sample (about 2.5 kg) was collected, wrapped in cloth bags & transported to the home to observe the traditional preservation practices. Non‐edible portions were separated and washed sufficiently to remove dirt & dust. Traditionally, the processing steps of wild melons are illustrated in Figure 1.

Figure 1 depicts the steps of traditional preservation techniques of wild melons. The wild melons were washed with water to prepare samples and cut into two halves horizontally or bilaterally. The sample was bilaterally cut for a salted dry product, not for cooked/curry purposes, followed by traditional techniques: sun drying and salting. The *Cucumis melo* var. *agrestis* was freshly collected from different farms at a mature and ripe stage. Samples were washed to remove dust, dirt, and fine hairs on the surface to improve product quality. After appropriate washing, wild melons were cut into two pieces using a stainless‐steel knife. Depending on the desire to utilize end products after drying, it was done in two ways. Horizontal cut (A) into two halves used after drying for cooked/curry purposes in the off‐season, while bilateral cut (B) halves with salt on their surface used before drying to consume after drying as snack foods, with a meal in the crunchy texture fried in oil with a sprinkling of spices. Cut wild melons were spread on the clean cloth under the scorching sunlight for 3–5 days for complete drying (moisture <10% on a dry basis). The observation was carried out after 1, 2, and 3 days.

### 2.3 Processing Methods

#### 2.3.1 Fresh Samples (Control)

The 500 g sample was packed in polyethene bags and appropriately labelled. It was stored in a freezer at −20±1^°^C for further analysis.

#### 2.3.2 Frying and Cooking

The 500 g of the sample was subjected to frying and 500 g for cooking with ingredients such as oil (50 mL), salt (2 g), red chilli powder (2 g), turmeric powder (0.5 g), onion (50 g chopped), tomato (50 g), garlic paste (2.5 g), and green chilli (10 g). The saucepan was placed on a medium‐level flame, and canola oil was added. The chopped onions were mixed and fried; after getting a golden brown colour, tomatoes, garlic paste, and remaining ingredients were mixed. Finally, the edible portion of the sample was mixed and cooked for 5 min to evaporate water on low flame. The cooked sample was cooled and placed in a freezer at −20±1°C for further analysis.

#### 2.3.3 Drying

The remaining 1 kg sample was divided into two halves and subjected to drying using a hot air dryer (70°C ± 2°C for 8–10 h up to 15–20% moisture content) and sun drying respectively (Azeem et al. 2021). Similarly, the samples were placed on a tray lined with butter paper at room temperature for 24 h for shade drying. After drying, samples were ground to get fine powder, passed via a 2 mm sieve, and poured into airtight containers for further analysis.

### 2.4 Proximate Composition

The AOAC method (2000) was followed to determine the moisture, crude fat, crude protein, ash, and available carbohydrates, whereas Mazumdar and Majumder (2021) and Asibey‐Berko and Tayie (1999) protocols were followed to determine crude fibre and energy value, respectively. For moisture content, a 5 g sample in a pre‐weighed empty petri dish was placed in an oven for 3–4 h at 70°C. The loss in weight was recorded as a measure of moisture content (on a dry weight basis). For crude fat, a sample (2 g) was poured in a majonnier tube, then 10 mL ethanol, 10 mL diethyl either, and 25 mL petroleum ether were added, shaken and left for 1 h. Later, the transparent solution was separated and dried at 100°C for 1 h, and this process was repeated three times. The conical flask was removed from the oven, cooled, and weighed with the sample. For protein, the mixture of 2 g sample, 30 mL sulphuric acid (Conc.), 2 g potassium sulphate, and 0.2 g copper sulphate in a Kjeldahl flask was heated for 6–8 h till colourless or light sky colour appeared, followed by cooling, and filtration. It was transferred to a 250 mL volumetric flask and made up with distilled water, followed by distillation and titration. The calculated nitrogen value was multiplied with a factor of 6.25 to get the protein percentage. The crude fibre was determined by taking a 2 g sample mixed with 1.25% H_2_SO_4_ and 1.25% NaOH. The sample was filtered with Whatman paper No. 4 and washed with hot distilled water, then with 1% HNO_3,_ and again with hot water. The final ash was weighed after burning the residue. The sample (5 g) was transferred to a pre‐weighed crucible and ignited in a muffle furnace at 550°C to obtain ash. The available carbohydrate content was calculated by estimating all the other fractions by proximate analysis, i.e. carbohydrate (%) = 100 − (% moisture + % ash + % fat +% protein +% fibre). The energy value (kcal/100 g) was calculated by using the formula of by multiplying fat, carbohydrate, and protein by 8.37, 3.57, and 2.44, respectively.

### 2.5 Mineral Analysis

For minerals analysis, 0.5 g of sample was taken in a conical flask, and 10 mL of nitric acid was added. The mixture was heated on a hot plate at 350–400°C for 1 h, and the flask was covered with a watch glass, followed by adding 2.0 mL more nitric acid and 3.0 mL of hydrogen peroxide and heating and cooling to obtain a colourless solution. It was filtered in a volumetric flask (25 mL) using Whatman paper No.42 and made up the final volume with nitric acid (0.1N). Finally, minerals were analysed through a Perkin Elmer atomic absorption spectrophotometer (AAS) (Kazi et al. 2010). Before analysis, 1, 2, 5, and 10 ppm standards of each mineral were run to get a calibration curve.

### 2.6 Vitamins

Vitamin B‐complex and vitamin C were determined followed by Marzougui et al. (2009) and Babarinde and Fabunmi (2009), and β‐ carotene was carried out by the methods of Siriamornpun et al. (2012) and Arlai (2009). Vitamin C (mg/100g) was also calculated using the titrimetric method described by Mazumdar and Majumder (2021).

### 2.7 Phytochemical Analysis

Phytochemicals such as alkaloids, tannins, saponins, flavonoids, and total phenols were quantified using established methods. Alkaloids were extracted and precipitated using the technique by Harborne (1993) and expressed in mg/g. Tannins were determined using Harborne (1993) and Ebrahimzadeh et al. (2008) methods, involving extraction followed by absorbance measurement. Saponins were extracted with ethanol and partitioned using diethyl ether and n‐butanol, as Obadoni and Ochuko (2002) described. Flavonoids were extracted with aqueous methanol and quantified following Boham and Kocipai (1994). Total phenols were measured using the Folin–Ciocalteu reagent according to the method by Mushtaq et al. (2015).

### 2.8 Statistical Analysis

Each experiment was repeated thrice, and results were presented as mean ± standard deviation and percentages using Microsoft Office Excel 2007. The numeric data generated was analysed using IBM SPSS Statistics 30 for analysis of variance (ANOVA). Duncan's multiple range test was applied to examine the means at a *p* < 0.05. Origin 2021 software (OriginLab, MA, USA) was used for principal component analysis.

## 3 Results and Discussion

### 3.1 Proximate Composition of Wild Melon/*Chibhar*

The results of the proximate composition of the wild melon are shown in Table 1. The moisture content varied amongst fresh, fried, cooked, thermally dehydrated, and sundried from 85.93%, 62,80%, 62.08%, 10.85%, and 11.64%, respectively. The highest ash content was recorded at 9.15% in the thermally dehydrated sample, followed by drying under sunlight (8.13), 4.05% ash in cooked samples, 3.97% in fried, and 2.69% in fresh samples. The crude fibre, fat, proteins, and carbohydrates were significantly (*p <* 0.05) highest in samples dried under sunlight and lowest in fresh samples. The estimation of energy values was calculated and found to be 45.76 kcal 100 g^−1^ in the fresh sample and was significantly (*p* < 0.05) highest in thermally dehydrated samples, i.e. 243.25 kcal 100 g^−1^. This non‐traditional vegetable is also a valuable source of energy and micronutrients in diets (Grivetti and Ogle 2000). Non‐traditional vegetables are safe for dietary consumption, and the high nutritional value of these vegetables could be utilized to combat malnutrition and associated conditions (Mih et al. 2017). The above results agree with the findings of Satter et al. (2016). The moisture percentage of some vegetables commonly consumed in Pakistan is similar to our findings (Hussain et al. 2011). Some studies by Hussain et al. (2010), Raju et al. (2018) and Traoré et al. (2017) found the impact of treatments on variation in moisture content.

**TABLE 1:** Proximate composition (%) of wild melon affected by different processing methods.

Treatments	Moisture	*CHO	*CF	Fat	*CP	Ash	Energy (Kcal 100 g ^−1^)
**Fresh**	85.93±2.162^a^	6.92±0.174^d^	7.97±0.200^c^	2.47±0.062^e^	2.25±0.056^d^	2.69±0.067^d^	45.76±1.152^e^
**Fried**	62.80±1.580^b^	16.28±0.409^c^	8.04±0.202^c^	3.47±0.087^d^	2.95±0.074^c^	3.97±0.099^c^	61.94±1.559^d^
**Cooked**	62.08±1.562^b^	16.60±0.417^c^	7.30±0.183^d^	4.53±0.114^b^	3.02±0.076^c^	4.05±0.101^c^	71.59±1.802^c^
**Thermally dehydrated**	10.85±0.273^c^	42.32±1.065^b^	9.15±0.230^b^	3.95±0.099^c^	18.85±0.474^b^	9.11±0.229^b^	243.25±6.122^a^
**Sun dried**	11.64±0.2929^c^	46.40±1.168^a^	10.23±0.257^a^	4.96±0.124^a^	16.79±0.422^a^	8.13±0.204^a^	228.74±5.757^b^

Values are expressed as mean standard deviation (*n* = 3); Mean values followed by the same letter down the column were not significantly different at *p* < 0.05 Duncan's multiple range test (DMRT); *CHO: Carbohydrate; * CF: Crude fibre; *CP: Crude protein.

According to AOAC (2000), a decrease in moisture value enhances the shelf‐life quality of food products. Our findings (Table 1) show that fresh wild melon contains high moisture content, which suggests that this melon in fresh form may not be of the same quality if stored; hence, it may deteriorate due to increased water activity helps maintain the protoplasmic contents of cells (Gbadamosi et al. 2011). Moreover, this condition is favourable for greater enzymatic and co‐enzyme activities needed for the metabolic mechanism of vegetables (Iheanacho and Udebuani 2009). However, elevated water activity may lead to bacterial spoilage during storage (Onyeike et al. 2003; Emebu and Anyika 2011). In earlier studies, non‐traditional edible plants' ash content has been reported between 0.65‐26.70% (Demir et al. 2006; Kagale and Sabale 2014; Kalita et al. 2014 and Tunçtürk and Özgökçe 2015). Roe et al. (2013) investigated that the ash content of wild plants was significantly higher than commercially available vegetables. An increased ash percentage as compared to commercial vegetables such as lettuce (0.4%) and spinach (0.7%) was found by Salazar et al. (2006) on a dry basis. Carbohydrates results were significantly (*p* < 0.05) higher than the already reported as compared to non‐traditional edible plants of Pakistan (Khan et al. 2013) and commercial vegetables (*Corchorus olitorius Amaranthus cruentus, Celusia argenta*) in Nigeria (Onwordi et al. 2009). The high levels contribute significantly (*p* < 0.05) to the energy value of food (Yirankinyuki et al. 2015). A diet lacking carbohydrates may result in poor mental function, tiredness or fatigue, and lack of endurance and stamina (Tardy et al. 2020), whereas high carbohydrates are a rich energy source and may be a proper tool for nourishment in rural people (Fanta and Neela 2019). Moreover, fibre also prevents weight, diabetes, constipation, risk of heart diseases, increased serum cholesterol, hypertension, breast/colon cancers etc. (Albuquerque et al. 2020). These findings resemble non‐traditional Pakistani edible plants and vegetables (Hussain et al. 2011; Shad et al. 2013; Vadivel and Janardhanan 2005). Belewu and Babalola (2009) reported that the crude fibres could be valuable if treated with microbes. Hussain et al. (2010), Hameed et al. (2008), and Aberoumand and Deokule (2009) assessed fibre content of 9.5–12.12% in selected non‐traditional plants, which is similar to our findings. It helps to decrease obesity risk and maintain a healthy digestive tract, reduce the risk of cancer and control blood sugar (Emebu and Anyika 2011; Maki et al. 2012; Gafar et al. 2011; Hasan and Umar 2006).

Barua et al. (2015) found a significant change in the crude protein in different plant samples. Hussain et al. (2009) reported the protein content of ginger; Shah et al. (2010) reported that protein‐rich plants usually contain 23–33% protein. Besides, our findings show a moderate protein level in non‐traditional plants, which differs from reported studies (Hussain et al. 2010; Hameed et al. 2008). Notably, these non‐traditional plants eaten as leafy vegetables showed significantly higher protein than cultivated leafy vegetables (Geeta and Sharma 2015). Umar et al. (2007) found 6.30% protein in water spinach, lower than in sweet potato leaves 24.85%. The high protein suggests their potential as a valuable protein source (Barua et al. 2015). Similarly, 0.21%‐ 0.45% fat in some Nigerian leafy vegetables (*Corchorus olitorius Amaranthus cruentus, Celusia argenta*) (Onwordi et al. 2009) and 2.00% and 3.01% fat in wild vegetables of Pakistan and Nigeria (Hussain et al. 2011; Khan et al. 2013 and Nkafamiya et al. 2010). Our results are aligned with those of Coşkun et al. 2004), Cherney and Cherney (2005), and Hussain et al. (2009). Ayuba et al. (2011) reported a crude fat content of 6% in roots and 15.52% in the seed of *Datura innoxia*. The lower fat content may help to prevent hyperlipidemia and reduce the risk of heart disease, cancer, and coronary artery damage (Onunogbu 2002).

### 3.2 Mineral Composition

The mineral composition of wild melon was analysed (Table 2). The results revealed that wild melon had copper, iron, zinc, manganese, calcium, magnesium, sodium, and potassium contents (7.71 mg 100g^−1^, 51.06 mg 100g^−1^, 10.46 mg 100g^−1^, 9.90 mg 100g^−1^, 403.92 mg 100g^−1^, 318.8 mg 100g^−1^, 27.71 mg 100g^−1^, and 758.64 mg 100g^−1^, respectively) were also significantly lesser in quantity in raw than fried and cooked/curry samples. In the case of the cooked/curry sample, the mineral elements were slightly higher than in fresh samples, whereas the dried samples (thermally dehydrated and shade‐dried samples) had the significantly (*p* < 0.05) highest value of mineral elements. The copper content showed a non‐significant difference between cooked/curry, thermally dehydrated, and shade‐dried samples.

**TABLE 2:** Influence of various processing methods on mineral composition (mg 100g^−1^) of wild melon.

Treatments	Copper	Iron	Zinc	Manganese	Calcium	Magnesium	Sodium	Potassium
**Fresh**	7.71±0.194^c^	51.06±1.285^d^	10.43±0.262^d^	9.90±0.249^d^	403.92±10.165^b^	381.8±9.608^d^	27.71±0.697^d^	758.64±19.092^c^
**Fry**	5.89±0.148^d^	54.89±1.381^c^	40.33±1.014^c^	34.22±0.861^b^	419.78±10.564^b^	792.00±19.932^a^	379.22±9.543^c^	1397±35.157^a^
**Cooked/curry**	11.44±0.287^a^	64.44±1.621^a^	58.11±1.462^b^	24.22±0.609^c^	420.89±10.592^b^	456.44±11.487^c^	626.44±15.765^a^	1090.89±27.453^b^
**Thermally dehydrated**	10.69±0.269^b^	59.71±1.437^b^	62.84±1.581^a^	35.18±0.885^b^	583.01±14.672^a^	573.79±14.440^b^	641.78±16.151^a^	681.85±17.160^d^
**Sun dried**	11.00±0.277^b^	59.90±1.507^b^	64.53±1.624^a^	37.09±0.9334^b^	602.35±15.159^a^	596.13±15.002^b^	549.32±13.824^b^	664.09±16.713^d^

Values are expressed as mean (n = 3); Values with different superscripts down the column significantly differ at *p< 0.05* DMRT.

Macro and micro‐minerals are very important in a recommended daily allowance (RDA) to achieve maximum health and production, as excess or lower amounts are harmful (Gaeta and Hider 2005). These results agreed with Agbaire and Emoyan (2012). Usually, minerals are not volatile; specifically, magnesium is involved in vital body processes and helps in cardiac and nerve function, protein synthesis, and energy metabolism (Insel et al. 2011). Moreover, mineral leaching was not supported due to slow water loss from the sample after sun drying (Hailu and Addis 2016). Additionally, processing conditions like peeling and boiling reduce iron and zinc content due to the removal of non‐edible parts. Similarly, the concept may significantly reduce the fried and peeled *D. abyssinica*. Moreover, peeled and fried tubers of *Dioscoreacayenensis* were reported to have a decrease in zinc content (Akin‐Idowu et al. 2009). However, a significant decline was studied when *A. esculentus*was cooked, where calcium was not affected by the sun drying (Hailu and Addis 2016).

### 3.3 Organic Acids

**FIGURE 2:**
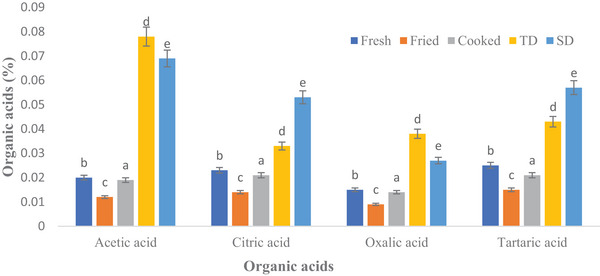
Influence of various processing methods on organic acid (%) in wild melon/chibhar (TD: thermally dehydrated and SD: sun‐dried).

Figure 2 presents the organic acids (acetic acid, citric acid, oxalic acid, and tartaric acid) in wild melon. The dried samples (thermally dehydrated and shade dried) were observed to have an increased number of organic acids compared to fresh, fried, and cooked/curry samples. Acetic acid, citric acid, oxalic acid, and tartaric acid were recorded in thermally dehydrated and shade‐dried samples as 0.078%, 0.033%, 0.038%, 0.043% and 0.069%, 0.053%, 0.027% and 0.057% respectively. The fresh and fried samples had values of 0.020%, 0.023%, 0.015%, 0.025% and 0.012%, 0.014%, 0.009%, and 0.015% for acetic acid, citric acid, oxalic acid, and tartaric acid, respectively. However, cooked samples had less effect and contained 0.019%, 0.021%, 0.014%, and 0.021% of acetic acid, citric acid, oxalic acid, and tartaric acid, respectively.

Non‐traditional veggies also include organic acids (e.g. citric acid, lactic acid, acetic acid, and tartaric acid), which are effective anti‐bacterial agents against psychrophilic and mesophilic microbes in fresh‐cut fruit and vegetables (Bari et al. 2003). Organic acids' antimicrobial activity is attributed to pH drop in the environment, interruption of membrane transport and/or permeability, anion buildup, or a decrease in internal cellular pH caused by the dissociation of hydrogen ions from acid (Beuchat 1999).

### 3.4 Vitamin Content of Wild Melon/*Chibhar*

The results of vitamin content of wild melon and different processing methods are shown in Table 3. The β‐ carotene content in fresh, thermally dehydrated, cooked/curry, shade‐dried, and fried samples was 105 IU, 103 IU, 99 IU, 101 IU, and 79 IU, respectively. Among water‐soluble vitamins, reductions in Vitamin C were noted in the fried sample (1.23 mg 100g^−1^) compared to the cooked/curry and fresh samples (2.53 mg 100g^−1^ and 2.87 mg 100g^−1^). Vitamins B_1_, B_2,_ and B_3_ in fresh, thermally dehydrated, cooked/curry, shade dried, and fried samples were found to be as 0.027, 0.024, 0.024, 0.023, 0.014 mg100g^−1^, 0.033, 0.032, 0.028, 0,031, 0.021 mg 100g^−1^ and in B3 0.098, 0.083, 0.079, 0.079 and 0.063 mg 100g^−1^, respectively. The level of vitamins B5, B6, and vitamin K in fresh, fried, cooked/curry, shade dried, and thermally dehydrated are 0.259 mg 100 g^−1^, 0.187 mg 100 g^−1^, 0.233 mg 100 g^−1^, 0.237 mg 100 g^−1^, 0.257 mg 100 g^−1^, and Vit B6 were 0.040 mg 100 g^−1^, 0.023 mg 100 g^−1^, 0.037 mg 100 g^−1^, 0.035 mg 100 g^−1^, 0.039 mg 100 g^−1^ and vitamin K reflected 16.4 mg 100 g^−1^, 12.7 mg 100 g^−1^, 14.3 mg 100 g^−1^, 15.9 mg 100 g^−1^ and 15.31 mg 100 g ^−1^respectively. The amount of vitamin B9 showed 7.89 µg 100 g^−1^, 5.19 µg 100 g^−1^, 7.83 µg 100 g^−1^, 7.62 µg 100 g^−1^, and 7.53 µg 100 g^−1^ in the thermally dehydrated sample, while vitamin B12 (cobalamine) was not detected in wild melon. The wild melon, kirir fruit, colocasia tubers, and colocasia leaves, analysed in their fresh, thermally dehydrated, curry, shade‐dried, and fried forms, were found to be rich sources of vitamins, with values closely aligning with those reported by Adeniyi et al. (2012). In various selected leafy vegetables, vitamins B₁, B₂, and B₃ have been identified as crucial for micronutrient metabolism, while vitamin C plays a significant role in collagen synthesis and protein metabolism (Vunchi et al. 2011). Additionally, β‐carotene & vitamin A are recognized as potential for maintaining eyesight (Johra et al. 2020). The reviewed literature and the present work highlighted that wild plants are good sources of riboflavin and ascorbic acid (Sajib et al. 2014).

**TABLE 3:** Influence of various processing methods on the vitamins content (mg 100 g^−1^) of wild melon.

Treatments	Beta‐carotene(IU)	Vitamin C	Vitamin B1	Vitamin B2	Vitamin B3	Vitamin B5	Vitamin B6	Vitamin B9	Vitamin B12	Vitamin K
Fresh	105±2.642^a^	2.87±0.072^a^	0.027±0.006^a^	0.033±0.008^a^	0.098±2.466^a^	0.259±0.006^a^	0.040±0.001^a^	7.89±0.198^a^	ND	16.4±0.4127^a^
Fried	79±1.9881^c^	1.23±0.031^d^	0.014±0.003^d^	0.021±0.005^d^	0.063±0.001^d^	0.187±0.004^c^	0.023±0.005^d^	5.19±0.130^c^	ND	12.7±0.3196^d^
Cooked/curry	99±2.491^b^	2.53±0.063^c^	0.021±0.005^c^	0.029±0.007^c^	0.078±0.001^c^	0.233±0.005^b^	0.037±0.009^b^	7.83±0.197^ab^	ND	14.3±0.359^c^
Sun dried	101±2.541^ab^	2.63±0.066^bc^	0.023±0.005^b^	0.031±0.007^b^	0.079±0.001^c^	0.237±0.005^b^	0.035±0.008^c^	7.62±0.191^ab^	ND	15.9±0.400^ab^
Thermally dehydrated	103±2.592^ab^	2.69±0.067_b_	0.024±0.006^b^	0.032±0.008^ab^	0.083±0.002^b^	0.257±0.006^a^	0.039±0.009^a^	7.53±0.1895^b^	ND	15.3±0.385^b^

*ND = not detected.

Values are expressed as mean ± standard deviation (*n* = 3); Values with different superscripts down the column significantly differ at *p* < 0.05 Duncan multiple range test (DMRT).

### 3.5 Phytochemical Content of Wild Melon

**FIGURE 3:**
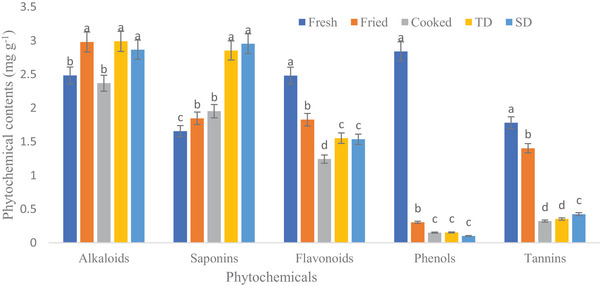
Influence of various processing methods on phytochemicals content (mg g^−1^) of wild melon (TD: thermally dehydrated and SD: sun‐dried).

Figure 3 shows the effect of processing methods on the phytochemical content of wild melon/chibhar. Total alkaloid content was significantly (*p* < 0.05) greater in the thermally dehydrated sample (2.989 mg g^−1^) followed by the fried sample (2.979 mg g^−1^), shade dried (2.865 mg g^−1^), fresh (2,483 mg g^−1^), and cooked/curry (2.365 mg g^−1^). Saponins content was significantly (*p* < 0.05) greater in shade dried sample (2.954 mg g^−1^) followed by the thermally dehydrated sample (2.853 mg g^−1^), cooked (1.952 mg g^−1^), fried (1.846 mg g^−1^) and in fresh it was noted as 1.655 mg g^−1^. Flavonoids, phenols, and tannins were significantly (*p* < 0.05) highest in fresh samples (2.48 mg g^−1^, 2.84 mg g^−1^, and 1.780 mg g^−1^) and lowest in treated samples. Flavonoids also serve as flavouring ingredients for spices and vegetables (Mensah 2023). Ndamitso et al. (2015) reported that water spinach leaves and stems contain flavonoids 0.52% and 0.34%, respectively.

### 3.6 Principal Component Analysis (PCA)

**FIGURE 4:**
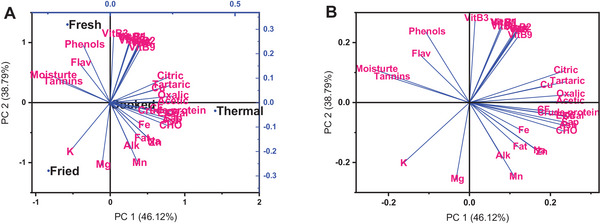
Biplot (A) and loading plot of the principal component analysis from PC1 and 2 of wild melon showing the association among variables.

This research used PCA to analyse wild melon's phytochemical contents and nutritional composition, which were subjected to different drying methods as it is an effective statistical method for detecting patterns and correlations in multidimensional datasets (Boateng and Yang 2021). Figure 4A,B illustrates the biplot and loading plots of the first two principal components, PC1 and PC2, representing 85 % of the total variability and according to Mamy et al. (2025), a cumulative PC ≥85.0% is sufficient to explain the dataset's total variance. As a result, we consider that PC1 and PC2 are appropriate for accurately discriminating scores between groups. The PC1 (46.12%) explained the sample variation the most. It also implies that PC1 explained the most connected characteristics (bioactive chemicals). In the biplot (Figure 4A), the cooked and thermal sample appeared on the positive side of PC1, whereas the fried and fresh samples appeared in the negative side of PC1 and this implies that wild melons can be classed based on the method of processing performed.

 The eigenvectors obtained via PCA provide information on the underlying causes that drive treatment variability. The eigenvectors reflect the coefficients of the PCs for each parameter, showing their contribution to the overall variability found in the dataset. The eigenvectors are extracted, and their implications are discussed (Table S1, Supporting Information). The PC1 coefficients for most parameters are positive, showing a positive relationship with this main component. Parameters with strong positive coefficients, such as saponin, oxalic acid, acetic acid, ash, and crude protein, substantially explain PC1 variance. This implies that changes in organic acids are the principal causes of variability among the samples. Parameters with negative coefficients, such as moisture, tannins, and phenols, showed a negative connection with the dorminant orgaionic acids (oxalic acid, acetic acid etc.) as shown in the loading plot (Figure 4B). This means that variations in moisture, tannins, and phenols are inversely proportional to the organic acids variability explained by PC1. The PC2 coefficients have both positive and negative values across different factors, showing a mixed influence on the variability explained by this main component. Parameters with significant positive coefficients, such as vitamins A, B1, B2, B3, B5, B9, and C, contribute positively to PC2 (Table S1, Supporting Information), implying that they are essential determinants causing variability orthogonal to PC1. Overall, the recovered eigenvectors provide helpful information about the interactions between phytochemical contents and nutritional composition in wild melon under different drying methods, and understanding these correlations can help optimize processing conditions and improve the nutritional and phytochemical properties of wild melon.

## 4 Conclusion

It is concluded that various processing techniques, including frying, cooking, thermal dehydration, and sun drying, significantly impact the nutritional composition and phytochemical content of wild melon (*Cucumis melo* var. *agrestis*) from Tharparkar, Sindh, Pakistan. Notably, moisture content decreased, and ash content increased significantly (*p* < 0.05) in thermally dehydrated samples. Sun‐dried samples exhibited the highest crude fibre, fat, protein, and carbohydrate values (*p* < 0.05). Additionally, mineral composition was significantly enhanced (*p* < 0.05) with drying techniques compared to fresh samples. beta‐carotene, C, and B varied significantly (*p* < 0.05) across fresh, fried, cooked, thermally dehydrated, and sun‐dried samples. Phytochemical analysis revealed significant variations (*p* < 0.05) in alkaloids, saponins, flavonoids, phenols, and tannins, with fresh samples having the highest levels. Organic acid analysis indicated higher levels of acetic, citric, oxalic, and tartaric acid in dried samples than in fresh and cooked samples. These findings suggest that drying techniques, such as sun drying and dehydration, can enhance wild melon's nutritional and phytochemical profile, highlighting its potential as a valuable food source. Further research is recommended to investigate the bioavailability of these essential nutrients.

## Author Contributions

**Saghir Ahmed Sheikh**: Conceptualization, investigation, writing–original draft, methodology, validation; formal analysis, data curation, project administration, visualization, software, supervision, resources. **Aasia Akbar Panhwar**: Data curation, formal analysis, software, methodology, writing–review and editing; visualization. **Parkash Meghwar**: Writing–review and editing; visualization. **Isaac Duah Boateng**: Writing–review and editing; software.

## Conflicts of Interest

No conflict of interest.

## Supporting information



Table 1. Eigen values for various bioactives and nutritional components on PC1 and 2.
