# Supplementary material for: Nutritional Composition and Phytochemical Changes in Wild Melon (Cucumis melo var. agrestis) Induced by Drying, Frying, and Cooking
Source: J Food Sci. 2025 May 19;90(5):e70229. doi: 10.1111/1750-3841.70229 (PMC12087523; doi:10.1111/1750-3841.70229)
Supplement: Supplementary file 1 — Table 1. Eigen values for various bioactives and nutritional components on PC1 and 2. [file JFDS-90-0-s001.docx]

Supplementary Table.

Table 1. Eigen values for various bioactives and nutritional components on PC1 and 2.

|  | PC1 | PC2 |
| --- | --- | --- |
| Moisture | -0.23304 | 0.10939 |
| CHO | 0.2364 | -0.09653 |
| CF | 0.17961 | -0.02698 |
| Fat | 0.1216 | -0.14926 |
| Crude protein | 0.24174 | -0.03572 |
| Ash | 0.24009 | -0.07783 |
| EKCal | 0.24492 | -0.04657 |
| Cu | 0.18715 | 0.05577 |
| Fe | 0.13096 | -0.09485 |
| Zn | 0.17541 | -0.1668 |
| Mn | 0.11191 | -0.24962 |
| Ca | 0.24202 | -0.04748 |
| Mg | -0.03311 | -0.25709 |
| Na | 0.16948 | -0.16234 |
| K | -0.16617 | -0.20512 |
| Acetic | 0.24591 | 0.00352 |
| Citric | 0.23471 | 0.1057 |
| Oxalic | 0.24697 | 0.02656 |
| Tartaric | 0.23869 | 0.06955 |
| VitA | 0.11724 | 0.24272 |
| VitC | 0.11837 | 0.24063 |
| Vit B1 | 0.08119 | 0.2651 |
| Vit B2 | 0.11603 | 0.24816 |
| VitB3 | 0.01398 | 0.27874 |
| VitB5 | 0.0813 | 0.26335 |
| VItB6 | 0.0807 | 0.25078 |
| VitB9 | 0.12181 | 0.22239 |
| VitK | 0.12104 | 0.24062 |
| Alk | 0.07959 | -0.17965 |
| Sap | 0.24538 | -0.0682 |
| Flav | -0.12251 | 0.15988 |
| Phenols | -0.10786 | 0.23445 |
| Tannins | -0.19466 | 0.08385 |
